# Supplementary material for: Infrared fingerprints of few-layer black phosphorus
Source: Nat Commun. 2017 Jan 6;8:14071. doi: 10.1038/ncomms14071 (PMC5227111; doi:10.1038/ncomms14071)
Supplement: Supplementary Information — Supplementary Figures, Supplementary Tables, Supplementary Notes and Supplementary References [file ncomms14071-s1.pdf]

Supplementary Figures

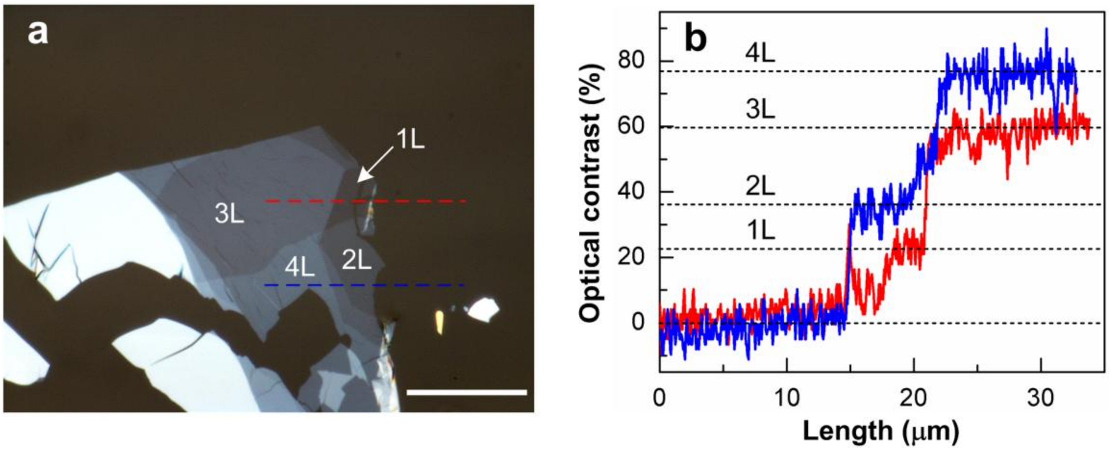

Supplementary Figure 1 | Thickness determination of BP flakes through optical contrasts. (a) Optical image of a representative few-layer BP sample with different thickness BP flakes present. The scale bar is 20 μm. (b) Optical contrast in the green CCD channel along the line cuts in (a). The contrast increases by ~20 % with each additional layer.

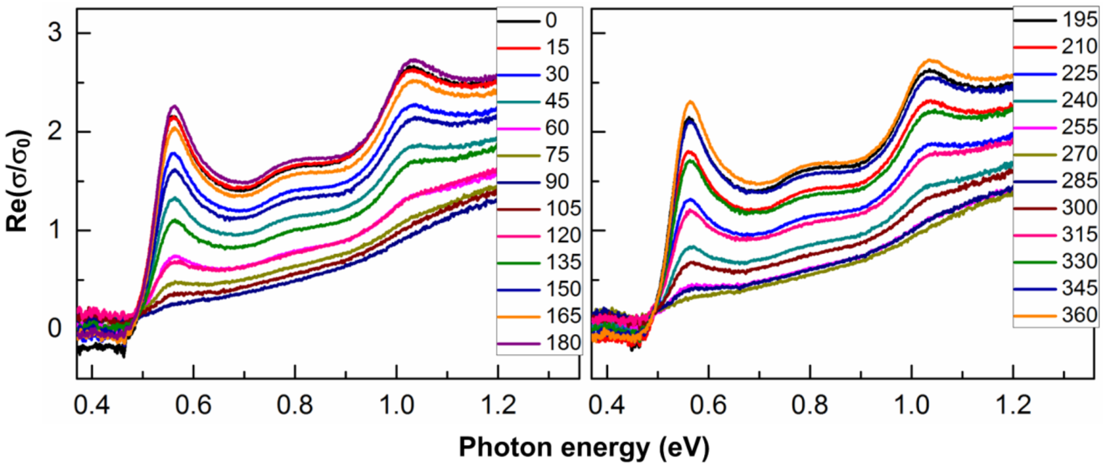

Supplementary Figure 2 | Polarization dependent IR spectroscopy. Real part of the optical conductivity σ for the 6L BP sample in Fig. 1b of the main

text, with the universal optical conductivity $\sigma_0 = \pi e^2/2h$ as the unit and the polarization angles of incident light from 0° to 360°.

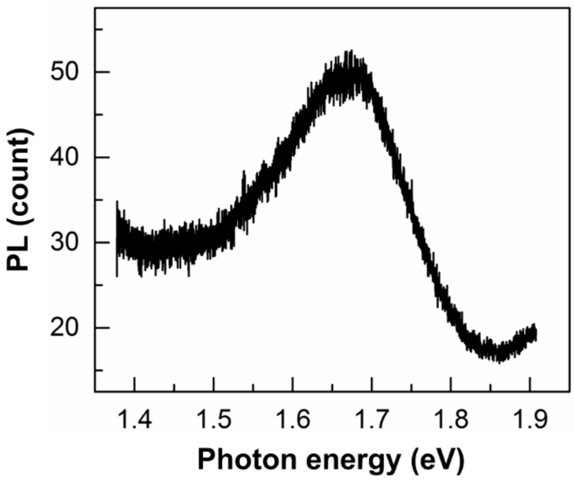

Supplementary Figure 3 | Photoluminescence spectrum of a monolayer BP. The monolayer was covered by a thin boron nitride (BN) layer for protection against degradation. The wavelength of the excitation laser is 514 nm. Monolayer BP has no feature in the IR spectral region accessible by our FTIR setup.

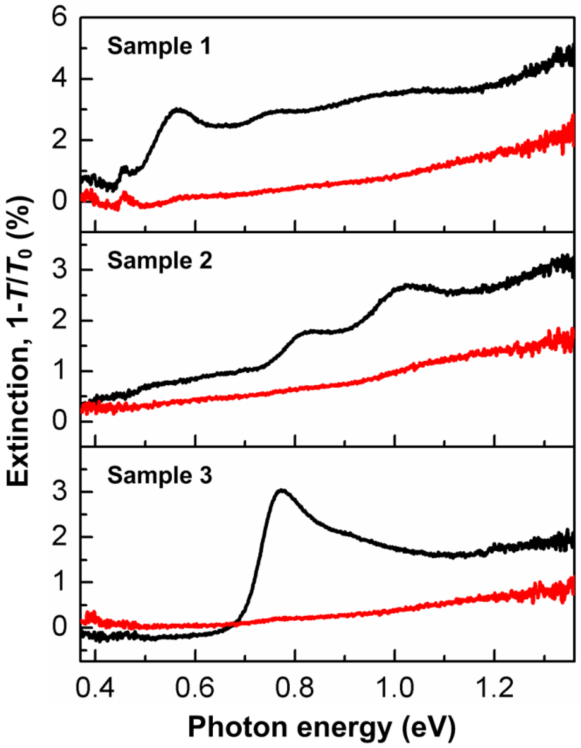

Supplementary Figure 4 | IR spectra for 3L BP flakes with different stacking orders. Black curves are measured with light polarization along armchair direction and red curves with polarization along zigzag direction. The spectra for sample 3 are also shown in Fig. 2b in the main article, in good agreement with the quasi-1D tight binding prediction. We come across atypical spectra as those for samples 1 and 2 in the figure occasionally, and attribute them to different stacking orders. These spectra show dramatic deviation from the normal one (sample 3). More studies will be carried out to assign the exact stacking order.

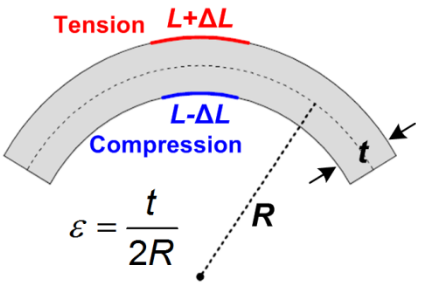

Supplementary Figure 5 | Strain setup. Schematic illustration of the two-point bending apparatus using a flexible polyethylene terephthalate (PET) substrate, tensile (compressive) strain can be introduced when the sample is placed on the top (bottom) surface of the PET substrate.

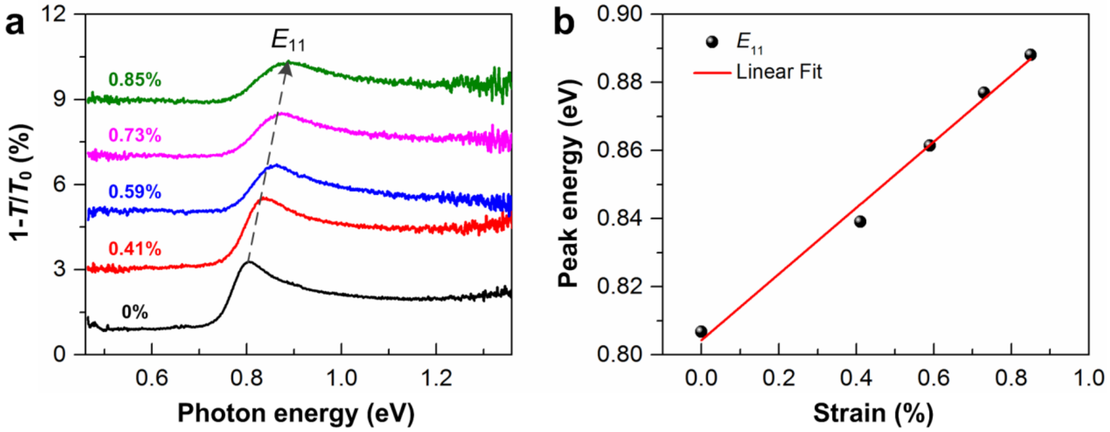

Supplementary Figure 6 | Electronic structure evolution of a 3L BP under tensile strain. (a) Extinction spectrum evolution with increasing tensile strain. The strain is applied along the armchair direction. The spectra are vertically offset for clarity. The incident light is polarized along the armchair direction. The dashed line traces the shift in the peak energy. (b) E_{11} peak energy as a function of uniaxial strain. The red line is a linear fit with slope 97 meV/%.

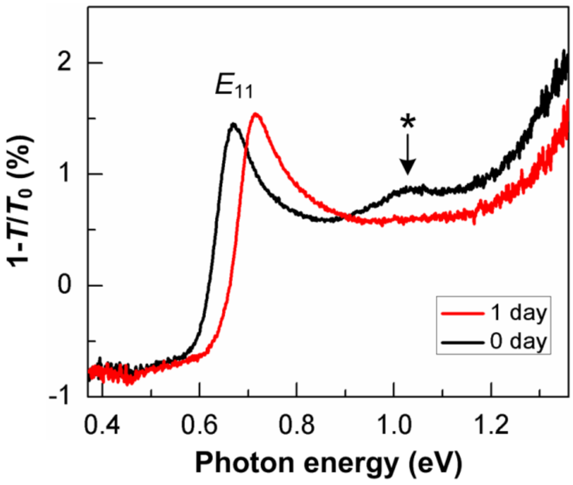

Supplementary Figure 7 | BP degradation in ambient condition. Extinction spectra (1-T/T_0) for the 4L BP sample in Fig. 2c of the main text, showing degradation after one day. E_{11} shifts to higher energy and the hybrid transition disappears. The marks E_{11} and asterisk (*) donate the first subband transition and hybrid transition, respectively.

Supplementary Note 1 | Quasi-1D tight binding model.

To get the band structure of bulk BP, one can start from the monolayer BP band structure and then take into account the layer-layer interactions. This is very similar to the case of 1D atomic chain, in which the energy dispersion can be obtained through the tight binding model. For few-layer BP, the same method can be adopted.

For simplicity, we consider only the nearest couplings for the conduction (γ_c) and valance bands (γ_v) respectively and only the Γ point ($k_x = k_y = 0$) at the Brillouin zone. We start with the Schrodinger equation of monolayer BP:

$$H_{1j}\varphi_{1j} = E_{1j}\varphi_{1j} \tag{S1}$$

where H_{1j} is the Hamiltonian at the Γ point of the Brillouin zone, E_{1j} is the eigenvalue, $j = (c, v)$, denoting the conduction and valance bands, respectively.

For bilayer BP, the Hamiltonian reads:

$$H_{2j} = \begin{pmatrix} H_{1j} & \gamma_j \\ \gamma_j & H_{1j} \end{pmatrix} \tag{S2}$$

The corresponding eigenvalues are $E_{2j} = E_{1j} \pm \gamma_j$, where γ_j is the nearest layer-layer coupling constant.

In a similar fashion, the Hamiltonian for N-layer BP can be described by a $N \times N$ matrix:

$$H_{Nj} = \begin{pmatrix} H_{1j} & \gamma_j & 0 & \cdots & 0 \\ \gamma_j & H_{1j} & \gamma_j & \cdots & 0 \\ 0 & \gamma_j & H_{1j} & \cdots & 0 \\ \vdots & \vdots & \vdots & \ddots & \vdots \\ 0 & 0 & 0 & \cdots & H_{1j} \end{pmatrix} \tag{S3}$$

The corresponding eigenvalues are $E_{Nj} = E_{1j} - 2\gamma_j \cos(\frac{n\pi}{N+1})$, where n = 1,2,3,...,N. Thus, the transition energy from valence subbands to conduction subbands with the same index n is given by

$$\begin{aligned} E_{nn}^{N} &= E_{\mathrm{c}} - E_{\mathrm{v}} \\ &= E_{\mathrm{g0}} - 2(\gamma_{\mathrm{c}} - \gamma_{\mathrm{v}})\cos(\frac{n\pi}{N+1}) \end{aligned} \quad \text{(S4)}$$

Where $E_{g0} = E_{1c} - E_{1v}$ is the bandgap of monolayer BP.

For $N >> n$, i.e. the layer number is large, equation (S4) can be expanded as Taylor series. By truncating at the first quadratic term of n, we have

$$\begin{aligned} E_{nn} &\approx E_{\mathrm{g0}} - 2(\gamma_{\mathrm{c}} - \gamma_{\mathrm{v}}) + \frac{(\gamma_{\mathrm{c}} - \gamma_{\mathrm{v}})\pi^2}{(N+1)^2} n^2 \\ &= a + b \cdot n^2 \end{aligned} \quad \text{(S5)}$$

where a and b are fitting parameters. We used equation (S5) to fit our 13L and 15L BP subband transition energies (Fig. 3b in the main text). The fitting parameters for the 13L: a = 0.38 eV, b = 0.039 eV, and for the 15L: a = 0.36 eV, b = 0.029 eV. From equation (S5), we see that parameter a is independent of layer number N, and $b \propto 1/(N+1)^2$. Our fitting parameters are consistent with these restrictions. It's worth noting, the n^2 dependence for the transition energies in equation (S5) can also be derived from an infinite quantum well model.

Supplementary Note 2 | Strain setup and strain calculation.

The two-point bending strain setup is schematically illustrated in Supplementary Fig. 5. Few-layer BP samples were first mechanically

exfoliated onto a polydimethylsiloxane (PDMS) sheet. Thin flakes are easy to identify by optical contrast. Subsequently, accurate determination of layer number and crystallographic orientation of the desired flake was achieved by polarized IR absorption spectra. The BP flake with well-defined orientation was then transferred to a flexible and transparent PET (polyethylene terephthalate) substrate. If we only planned to test strain effect on one specific direction (ZZ or AC directions), we typically used rectangular shaped PET stripe with dimensions of 4 cm x 1 cm x 0.3 cm. For the data shown in Fig. 4 in the main text, strain in both ZZ and AC directions was applied consecutively. In this case, the BP sample was sitting on a square shaped PET substrate with dimensions of 4 cm x 4 cm x 0.3 cm. Special care was taken to position the BP flake as close as possible to the center of the PET substrate, for accurate determination of strain values. Finally, the PET substrate holding BP flakes was mounted into the two-point bending apparatus. Through bending the PET substrate, uniaxial strain was applied to BP flakes due to van der Waals attraction. Tensile (compressive) strain can be achieved when the sample is located on the top (bottom) surface of the PET substrate. The applied strain ε is given by $\varepsilon = t/2R$, t and R are the thickness and radius of curvature of the bent substrate, respectively[S1].

Supplementary Table 1 | Transition energies of BP with different layer number.

Layer number	IR Absorption (eV)				DFT calculations[a] (eV)
	E_{11}	E_{22}	E_{33}	E_{44}	Quasiparticle gap
1	1.67[b]				2.00
2	1.14				1.32
3	0.78				1.06
4	0.67				0.92
5	0.63	1.30			0.83
6	0.56	1.03			0.76
7	0.51	0.96			0.71
8	0.48	0.85			0.67
9	0.47	0.77	1.19		0.64
13	0.40	0.54	0.74	0.98	0.56
15	0.38	0.48	0.63	0.82	0.54
Bulk	0.34				0.30

[a]Data from Ref[S2]

[b]Data is extracted from the PL spectroscopy.

Supplementary References

S1 Ugural, A. C. *Mechanics of Materials*. (Wiley: New York, 2008).

S2 Tran, V., Soklaski, R., Liang, Y. & Yang, L. Layer-controlled band gap and anisotropic excitons in few-layer black phosphorus. *Phys. Rev. B* **89**, 235319 (2014).
